# The role of noncoding RNAs in metabolic reprogramming of cancer cells

**DOI:** 10.1186/s11658-023-00447-8

**Published:** 2023-05-09

**Authors:** Amir Safi, Mohammadreza Saberiyan, Mohammad-Javad Sanaei, Samaneh Adelian, Fateme Davarani Asl, Mahsa Zeinaly, Mahdi Shamsi, Reza Ahmadi

**Affiliations:** 1 Clinical Biochemistry Research Center, Basic Health Sciences Institute, Shahrekord University of Medical Sciences, Shahrekord, Iran; 2 Cellular and Molecular Research Center, Basic Health Sciences Institute, Shahrekord University of Medical Sciences, Shahrekord, Iran; 3 Department of Hematology and Blood Banking, School of Allied Medical Sciences, Shahid Beheshti University of Medical Sciences, Tehran, Iran; 4 Department of Biology, Faculty of Sciences, University of Guilan, Rasht, Iran; 5 Department of Cell and Molecular Biology, Najafabad Branch, Islamic Azad University, Najafabad, Iran; 6 Medical Plants Research Center, Basic Health Sciences Institute, Shahrekord University of Medical Sciences, Rahmatiyeh Region, Shahrekord, Iran

**Keywords:** Cancer, Metabolic reprogramming, Noncoding RNAs, MicroRNA, Long noncoding RNA, Circular RNA

## Abstract

Metabolic reprogramming is a well-known feature of cancer that allows malignant cells to alter metabolic reactions and nutrient uptake, thereby promoting tumor growth and spread. It has been discovered that noncoding RNAs (ncRNAs), including microRNA (miRNA), long noncoding RNA (lncRNA), and circular RNA (circRNA), have a role in a variety of biological functions, control physiologic and developmental processes, and even influence disease. They have been recognized in numerous cancer types as tumor suppressors and oncogenic agents. The role of ncRNAs in the metabolic reprogramming of cancer cells has recently been noticed. We examine this subject, with an emphasis on the metabolism of glucose, lipids, and amino acids, and highlight the therapeutic use of targeting ncRNAs in cancer treatment.

## Introduction

The noncoding RNAs (ncRNAs) are untranslated transcripts, which are classified as short (19–31 nucleotides), mid (20–200 nucleotides), and long (> 200 nucleotides) based on their length. Among them, the most extensively studied in cancer are microRNAs (miRNAs), which belong to the short ncRNAs class (22–25 nucleotides in length) and long-ncRNAs (lncRNAs), which represent the largest class of noncoding transcripts, with about 55,000 genes along the human genome[1]. NcRNAs can affect cell fate and survival through various mechanisms, including transcriptional and posttranscriptional modification, chromatin remodeling, and signal transduction. They present a tissue-specific expression pattern; are highly dysregulated in cancer; and are considered promising diagnostic, prognostic, and therapeutic targets [2].

**Fig. 1:**
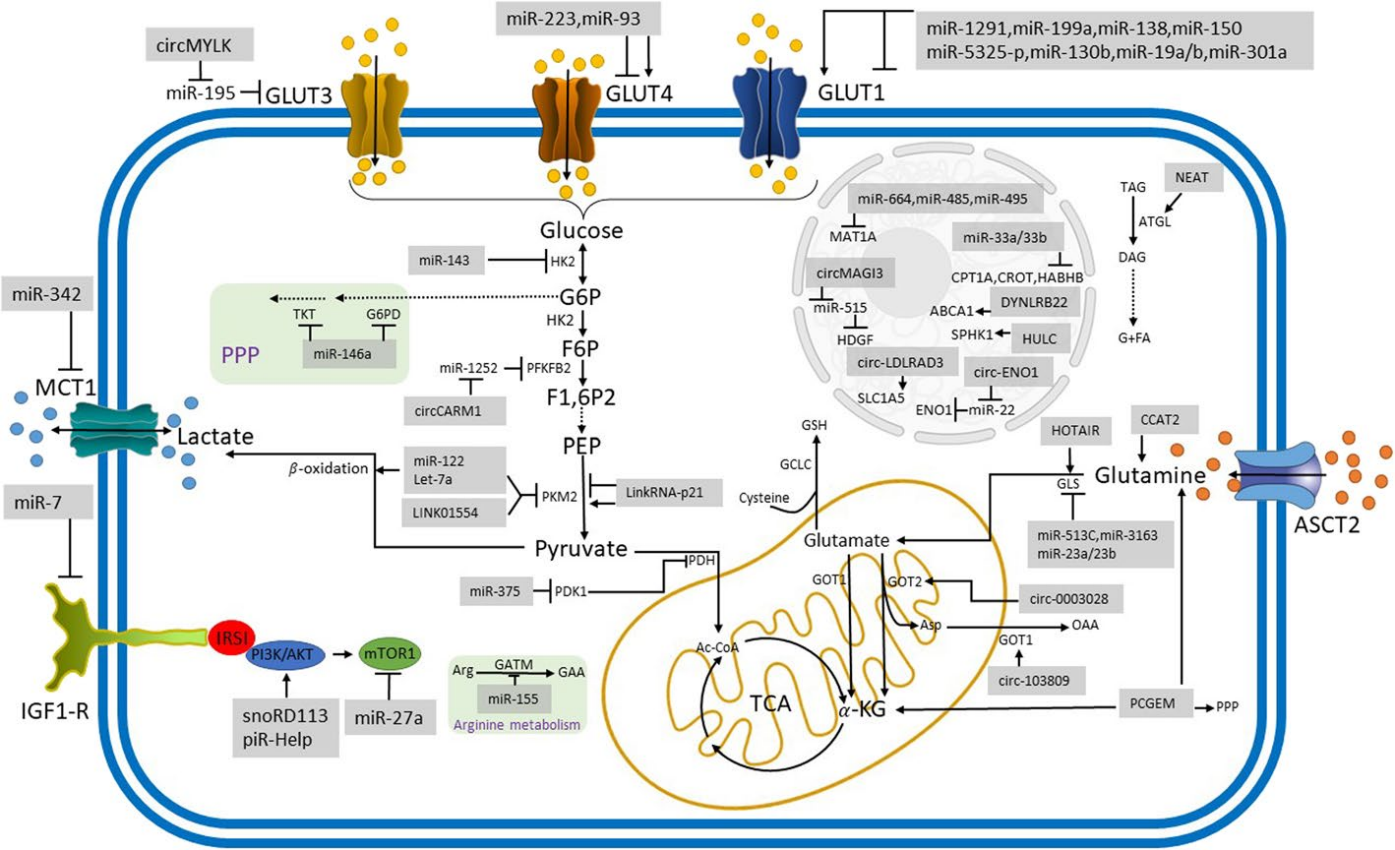
Noncoding RNAs regulate metabolism in cancer cells. MCT1: monocarboxylate transporter 1; GLUT: glucose transporter; ASCT2: alanine, serine, cysteine, and glutamate transporter; IGF1-R: insulin like growth factor1 receptor; G6P: glucose-6-phosphate; PEP: phosphoenolpyruvate; HK2: hexokinase 2; PFKFB2: 6-phosphofructo-2-kinase/fructose-2,6-biphosphatase 2; PKM2: pyruvate kinase isozyme M2; PDH: pyruvate dehydrogenase; PDK1: pyruvate dehydrogenase kinase 1; G6PD: glucose-6-phosphate-dehydrogenase; TKT: transketolase; IRS1: insulin receptor substrate 1; GAA: glutamic acid; GATM: glycine amidinotransferase; Ac-CoA: acetyl-CoA; GOT1: glutamate–oxaloacetate transaminase 1; α-KG: alpha-ketoglutarate; GSH: glutathione; GCLC: glutamate-cysteine arginine ligase catalytic subunit; GLS: glutaminase; MAT1A: methionine adenosyltransferase 1A; CPT1A: carnitine palmitoyltransferase 1A; CROT: carnitine *O*-octanoyltransferase; HADHB: hydroxyacyl-CoA dehydrogenase trifunctional multienzyme complex subunit beta; ABCA1: ATP binding cassette subfamily A member 1; SPHK1: sphingosine kinase 1; TAG: triacylglycerol; DAG: diacylglycerol; G: glycerol; FA: fatty acid; IRS1: insulin receptor substrate 1; Arg: arginine; GAA: glutamic acid; GATM: glycine amidinotransferase; Ac-CoA: acetyl-CoA; GOT1: glutamate–oxaloacetate transaminase 1; GOT2: glutamate–oxaloacetate transaminase 2; α-KG: alpha-ketoglutarate; Asp: aspartate; OAA: oxaloacetate; GLS: glutaminase

Recent evidence reported that miRNAs might involve up to 60% of human genome regulation. MiRNAs regulate many essential biological functions critical to normal development, with the deregulation of these same miRNAs later in life contributing to developing diseases such as cancer. In cancer, miRNA expression and function may be tissue and cell specific, with miRNAs serving as tumor suppressors, oncogenes, or in some cases, both [3]. Changes in the level of lncRNAs have been identified as one of the measurable markers in most cancers and, in many cases, are involved in causing complications of the disease. Examination of the functional pathways of lncRNAs has shown that lncRNAs have an active role by interacting with proteins, chromatins, and other RNAs in different paths of cell growth, proliferation, differentiation, migration, apoptosis, and cell death [4]. Other types of ncRNA, such as PIWI-interacting RNA (piRNA), small nucleolar RNAs (snoRNAs), and circular RNAs (circRNAs), exist in addition to miRNAs and lncRNAs, and are involved in a variety of gene expression control processes at the transcription, posttranscriptional, and translational stages. Among these categories of ncRNAs, circRNAs, another form of lncRNAs, have been found in different cancers. It has been clarified that circRNAs can regulate gene expression at protein and RNA levels, directly affecting cellular processes such as cell cycle, proliferation, epithelial–mesenchymal transition, and cancer progression [5]. Metabolic reprogramming is one of the most significant characteristics of tumors, which are heterogeneous and malignant diseases. According to mounting evidence, cancer metabolism contributes significantly to cancer signaling that maintains tumorigenesis and survival and its broader implications for controlling the antitumor immune response by influencing the expression of immune molecules and releasing metabolites [6]. The main aim of metabolic reprogramming in cancer cells is to balance the energy expenditure and facilitate biomass synthesis to support cancer cell proliferation. Hypoxia and acidosis are key characteristics of the tumor microenvironment, which enhance the immune tolerance in the tumor microenvironment by inducing tumor-promoting phenotypes of stromal cells and repressing the antitumor capacity of infiltrating immune cells [7, 8]. Thus, the secondary objective of metabolic reprogramming in cancer is to reshape the tumor microenvironment. There is some evidence that ncRNA plays an important role in metabolic reprogramming in cancer cells as well as regulating feedback between changes in energy signaling and ncRNA expression or activity. Studies reveal that ncRNAs are involved in the regulation of glucose metabolism, glycolysis, the pentose phosphate pathway (PPP), the tricarboxylic acid (TCA) cycle, amino acid metabolism, and redox balancing, fatty acid (FA) metabolism [9] (Fig. 1).

In this study, we aimed to review the role of ncRNA in the metabolic reprogramming of cancer cells.

## MiRNAs regulate cancer metabolic reprogramming

Cancer cells can elevate their survival potential, particularly in critical circumstances, through increased nutrition taking and reprogramming different metabolic pathways. Among various types of ncRNAs, the contribution of miRNAs to metabolic gene regulation is well investigated at posttranscription levels [10]. MiRNAs, like the other ncRNAs, play pivotal roles in reprogramming different metabolic pathways; for instance, in recent years, the role of miR-19a/19b in glucose metabolism [11], miR-21 in amino acid metabolism [12], and miR-33a/33b in lipid metabolism have been accurately described [13].

Glucose is the primary carbon source in the cell, and its homeostasis must be preserved during the metabolic reprogramming of cancer cells for survival in critical conditions as a staple cellular fuel. Meanwhile, miRNAs affect glucose homeostasis by regulating factors that provide and consume glucose. Insulin is the primary regulatory factor in glucose delivery to human cells [14]. Obviously, with any dysregulation in insulin routes, the fuel supply will be disrupted and the cellular energy will decline drastically. There is numerous evidence indicating that miRNAs regulate glucose metabolism via direct and indirect impacts on insulin pathways [15]. For example, miR-7 is represented as a tumor suppressor miRNA, which targets the 3′-untranslated region (UTR) of the insulin-like growth factor receptor gene directly in glioblastoma cells and, consequently, suppresses glycolysis and cell growth [16].

Interestingly, several miRNAs have been identified as responsible for regulating glucose transporter (GLUT) expression in cancer cells [17, 18]. For example, GLUT1 expression in renal carcinoma cells is inhibited through the tumor suppressor activity of miR-1291 [19, 20]. It has been shown that miR-10b targeted components of the insulin signaling pathway. The overexpression of miR-10b led to chemoresistance in colorectal cancer cells by the involvement of the GLUTs, especially GLUT2 and GLUT3, with high glucose affinity [21, 22].

Epigenetic alterations are also one of the mechanisms frequently observed in glioblastoma and are effective in metabolic reprogramming. For example, histone deacetylase is associated with the metabolism of cancer cells and the progression of glioblastoma. In the study of Kwak et al. (2022), the knockdown of histone deacetylase 2 was associated with the upregulation of miR-3189 and decreased GLUT3 expression. In addition, the knockdown of histone deacetylase 2 expressions in glioblastoma cells showed that it leads to the induction of cell death by inhibiting GLUT3. Through the reprogramming of glucose metabolism by regulating miR-3189-inhibited GLUT3 expression, their results showed the critical function of histone deacetylase 2 in the development of glioblastoma tumors and provided a possible novel therapeutic approach for the treatment of glioblastoma multiforme (GBM) [23].

Although miR-let-7 is one of the oldest known miRNAs, new investigations still focus on its role in various cancers. Shi et al. (2020) showed that miR-let-7a-5p as a tumor suppressor leads to the inhibition of GLUT12. GLUT12, with its role in regulating anaerobic glycolysis, leads to the induction of metastasis and tumor growth in triple-negative breast cancer (TNBC). Mechanistically, inhibiting GLUT12 causes changes in lactate production, glucose uptake, oxygen consumption rate, ATP production, and extracellular acidification rate. In addition, their findings clarified that while the high expression of let-7a-5p is associated with better clinical conditions in TNBC patients, while an increase in GLUT12 expression has the opposite effect. These findings suggest that the let-7a-5p/GLUT12 axis plays a significant role in TNBC tumor growth and metastasis, as well as aerobic glycolysis, and may be a potential treatment target [24]. The role of let-7 in metabolic reprogramming in correlation with various cancers was well described in the study of Li et al. [25].

MiR-233 and miR-133 directly regulate GLUT4, two further instances of miRNAs that control glucose uptake. It is interesting to note that miR-21 and miR-23a control two GLUT4 translocators, PTEN and SMAD4, and as a result, indirectly control the expression of GLUT4. Moreover, the expression of GLUT4 is controlled directly or indirectly by miR-21a-5p, miR-29a-3p, miR-29c-3p, miR-93-5p, miR-106b-5p, miR-133a-3p, miR-133b-3p, miR-222-3p, and miR-223-3p [26].

Cancer cells still have OXPHOS activity but prefer to produce ATP via aerobic glycolysis. The growth of cancer is more suited to glycolysis. Cancer tissues proliferate more quickly than healthy tissues, so in addition to energy, they require metabolic intermediates for the biosynthesis of macromolecules. It is possible to synthesize a variety of macromolecules, including nucleic acids, lipids, and proteins, which are necessary for the growth and proliferation of cancer, using intermediates from glycolysis and the truncated TCA cycle [27]. According to recent evidence, the switch of oxidative phosphorylation to glycolysis in cancer cells is regulated directly and indirectly by miRNA regulatory function. Indeed, by targeting the expression of metabolic enzymes involved in these pathways, miRNAs exert their effect on reprogramming glucose metabolism in cancer cells. A dual role for miRNAs in glucose metabolism in cancer cells has been described. Some miRNAs reduce glucose metabolism with a tumor suppressor role [28]. For example, miR-143 decreases the glucose metabolic rate by inhibiting hexokinase 2 expression (HK2, which catalyzes the phosphorylation of d-glucose and d-fructose to glucose 6-phosphate and fructose 6-phosphate), and as a result, prevents the proliferation and growth of cancer cells [29].

Conversely, it was shown that miR-155 suppresses miR-143, thus resulting in the upregulation of HK2 expression at the posttranscriptional level. Therefore, it was suggested that miR-155 could be responsible for the altered metabolism and motile and invasive abilities observed in breast cancer cells [30]. On the other hand, miR-122 promotes metastasis in breast cancer cells by inhibiting the expression of pyruvate kinase M2 (PKM2, the enzyme that regulates the glycolysis rate) [31]. A strategy cancer cells apply to elevate the glycolysis rate is to control the active enzymes in the pyruvate cycle, pyruvate dehydrogenase complex component X (PDHX) and pyruvate dehydrogenase complex (PDK). It has been reported that miR-375 ensures the survival of gastric carcinoma cells by targeting PDK1 [32]. Likewise, miR-26a has a similar function in colorectal cancer cells by inhibiting the expression of PDHX [33]. Also, several studies have clarified the role of miR-34a in targeting different glycolytic enzymes, including lactate dehydrogenase A (LDHA), HKs, PDK1, and glucose-6-phosphate isomerase (GPI) [34, 35]. In addition, some miRNAs are responsible for regulating glucose-related signaling pathways. For example, miR-218, as a tumor suppressor, is downregulated in oral cancer targeting the mTOR signaling pathway [36]. Moreover, miR-451 helps glioma cells adapt to metabolic stress conditions by modulating the AMPK/LKB1 pathway [37].

Cancer cells, as mentioned earlier, rely on glycolysis rather than oxidative phosphorylation to obtain their energy. The mitochondrial genome encodes 13 components of respiratory chain proteins required for oxidative phosphorylation [38]. Zhuang et al. found low-level expression of these components at mRNA and protein levels, including the cytochrome B (*mt-CYB*) and cytochrome C oxidase II (*mt-CO2*) genes in human hepatocellular carcinoma (HCC) tissues. Through mitochondrial gene sequencing, they identified significant abnormalities in the sequence and expression of mitochondrial miRNAs (mitomiRs). Bioinformatic analysis revealed that miRNA-181a-5p potentially suppressed mt-CYB and mt-CO2 expression. Therefore, overexpression of miRNA-181a-5p significantly reduces mt-CYB and mt-CO2 expression in HCC cells. In contrast, HK2 and GLUT1 are upregulated and associated with enhanced lactic acid release and elevated lactate dehydrogenase activity. They concluded that mitomiR-181a-5p is involved in glucose metabolism reprogramming, the promotion of tumor progression, and lung cancer metastasis in the early stage of liver cancer [39].

The tumor suppressor miRNA miR-200c is primarily known for its vital role in cancer stemness and epithelial phenotype. Based on the study of Chao et al. (2021), miR-200c was found to affect p53 regulation: with p53 deficiency, its expression is reduced and the epithelial–mesenchymal transition (EMT) and stem cell-like phenotype is induced, which will ultimately lead to the development of breast cancer. Knockout miR-200c, with the help of CRISPR, leads to p53 pseudo-mutation conditions. In addition to inducing EMT and stemness phenotype, it also causes cell reprogramming. To justify the mechanism of the miR-200c effect, it was found that the decrease in miR-200c expression and the mutation in p53 are associated with the reduction of phosphoenolpyruvate carboxykinase 2 (PCK2) expression. A decreased PCK2 expression in breast epithelial cells weakens oxidative phosphorylation and increases stemness. PCK2 is clinically associated with poor overall survival in breast cancer patients [40].

Notably, some studies highlighted the role of exosomes in the glucose reprogramming of human adult cells during tumorigenesis. Generally, miR-155 expression leads to the upregulation of glycolysis and glucose metabolism. In addition, miR-210 overexpression reduces oxidative phosphorylation rate in nonhypoxia conditions. According to Shu et al., these miRNAs were found in human melanoma-derived exosomes (HMEX) from all six melanoma cell lines, and were influential in increasing glycolysis and blocking oxidative phosphorylation in tumor cells. Transfection of miRNA inhibitors into HMEX inhibited the activities of miR-155 and miR-210, reversing the exosome-induced metabolic reprogramming of human adult dermal fibroblasts (HADF) [41].

Moreover, miRNAs from the let-7 family, particularly let-7 g, inhibited aerobic glycolysis in human HCC cancer cell lines. Let-7 overexpression inhibits glucose absorption in the HCC cancer studied, and let-7 notably reduces the expression of pyruvate dehydrogenase kinase isozyme 1 (PDK1) but not other oxidative phosphorylation enzymes [42]. In another study, Barisciano et al. studied the role of miR-27a on metabolic reprogramming and chemoresistance in colorectal cancer. They revealed the association of miR-27a overexpression with impaired oxidative phosphorylation and mitochondrial activities. According to their findings, miR-27a inhibited AMP-activated protein kinase, increased mTOR signaling, and collaborated with oncogenes and tumor cell metabolic regulators to create an aerobic glycolytic metabolism that supported biomass generation, unrestrained growth, and chemoresistance. They also verified this latter link in their patient cohort and cell line investigations [43].

The monocarboxylate transporter 1 (MCT1) is a crucial lactate transporter that regulates lactate flux between glycolytic cells and oxidative tumor cells. There is numerous evidence highlighting the over activity of MCT1 and its correlation with poor prognosis in different malignancies. Conversely, miR-342-5p is a tumor suppressor miRNA. Its exogenous overexpression is associated with apoptosis induction, reduced proliferation, and increased response to chemotherapy agents in various cancers such as non-small cell lung cancer, prostate, breast, cervical, colorectal, and HCC. miR-342-5p contributed to metabolism reprogramming, such as directly targeting MCT1 and regulating its posttranscription expression [44]. Cordoba et al. revealed that exogenous expression of miR-342-5p decreased MCT1 expression, disrupting metabolic flux and enhancing glucose consumption rather than lactate in cancer cells, and consequently, energy stabilization and a decline in the cell proliferation rate. It was recently shown that miR-342-3p controlled glycolysis by modifying glucose uptake, lactate production, and the extracellular acidification rate in hepatoma cells by blocking the IGF-1R-mediated PI3K/AKT/GLUT1 signaling pathway.

Interestingly, alterations in tumor metabolism not only meet the required energy for tumor cells but also impart immune response debilitation. The metabolic interdependence of tumor and immune cells causes metabolic conflict, restricting tumor-specific immune cell growth and activity. Moreover, high lactate levels suppress the division and activity of immune cells, including natural killer cells and lymphocyte T cells. According to these conclusions, Cordoba’s team discovered a substantial downregulation of immune-related pathways, as seen by lower immunophenoscores and cytolytic activity in tumors over-expressing miR-342-3p with a higher glycolytic score [45].

Cancer cells interact highly with other cells and their common niche for compatibility with adverse environmental conditions. In addition to intracellular miRNAs, recent studies focused on the impact of extracellular miRNAs on cancer cell reprogramming. Yan et al. identified an extracellular vesicle containing miR-105 derived from cancer cells through c-Myc inducement. This vesicle induces metabolic reprogramming by activating c-Myc signaling in cancer-associated fibroblasts (CAFs), indicating different metabolic programs for adapting to adverse metabolic environments. Under excellent resource conditions, miR-105-reprogrammed CAFs boost glucose and glutamine metabolism to fuel neighboring cancer cells. When nutrition levels are low and metabolic byproducts build up, these CAFs detoxify metabolic wastes such as lactic acid and ammonium by turning them into energy-rich metabolites. Therefore, miR-105-mediated stromal cell metabolic reprogramming promotes tumor progression by training the shared metabolic environment [46].

Due to its vital role in cell processes, lipid metabolism is highly regulated in normal cells. On the other hand, cancer cells reprogram lipid metabolism to be consistent with critical conditions. Therefore, cancer cells synthase lipid de novo instead of from exogenous sources [47]. Moreover, they prefer storing lipids to lipid oxidation for cell usage. They can also form cholesterol esters from free cholesterol to accelerate cancer cell migration. There are multiple metabolic enzymes involved in lipid metabolism. Tumor cells can reprogram lipid metabolism by ncRNAs, particularly miRNAs, by directly targeting these enzymes. For instance, fatty acid catabolism is inhibited by miR-33 activity, which directly suppresses the translation of enzymes in fatty acid β-oxidation such as carnitine palmitoyl transferase 1A (CPT1A) [48]. Moreover, the process of mitochondrial fatty acid oxidation is inhibited by a miRNA cluster activity, miR-199-miR-214, which targets myocardial peroxisome proliferator-activated receptor δ (PPARδ) during hypoxia conditions [49].

Cholesterol metabolism is one of the most crucial parts of lipid metabolism in cells, especially in hepatic cells. The abundance of miR-122 in liver cells (more than 70% of total miRNAs) highlights its critical regulatory role in cholesterol metabolism, such that miR-122 inhibition in mice leads to enhancement of hepatic fatty acid oxidation and a decrease in cholesterol synthesis. MiR-122 can also indirectly increase cholesterol synthesis enzymes such as 3-hydroxy-3-methylglutaryl-CoA synthase 1 (HMGCS1), 7-dehydrocholesterol reductase (DHCR7), 3-hydroxy-3-methylglutaryl-CoA reductase (HMGCR), and squalene epoxidase (SQLE) [50, 51].

Cruz-Gil et al. used miR-19b-1 to regulate abnormal acyl-CoA synthetase/stearoyl-CoA desaturase (ACSL/SCD). MiR-19b-1 could hinder the invasion of colon cancer cells by maintaining the ACSL/SCD metabolic axis [52]. The master regulator of lipid metabolism, peroxisome proliferator-activated receptor (PPARα), is a nuclear receptor that functions as a ligand-activated transcription factor. PPARα activates numerous enzymatic pathways in FA uptake, intracellular transport, FA activation and β-oxidation, and lipoprotein/cholesterol metabolism. Interestingly, PPARα is a known target of miR-21, an established oncogenic miRNA commonly upregulated in many solid tumors [53]. Azizi et al. indicated that the effect of miR-21 was impeded by the suppression of CD36, which suggests miR-21 acts on lipid metabolism through CD36, with the involvement of peroxisome proliferator-activated receptor gamma coactivator 1-beta (PPARGC1B) [54].

In general, the metabolism of sphingolipid is a crucial topic in cancer studies. Esnolipid metabolites, including sphingosine-1-phosphate (S1P) and ceramide, are vital in cell growth and apoptosis. Sphingosine kinase 1 (SPHK1) is an enzyme responsible for catalyzing the phosphorylation of sphingosine to SP1P and, as a result, moves the cell toward division and survival. Arora et al. showed that miR-495-3p directly targets SPHK1 and acts as a tumor suppressor of this enzyme in cell division, lactate dehydrogenase A (LDHA) activity, and cell colony formation in the cell. In addition, it became clear that inhibition of SPHK1 through miR-495-3p leads to mitochondrial dysfunction. The upregulation of miR-495-3p decreases mitochondrial energy homeostasis, increases reactive oxygen species (ROS) production, and induces apoptosis. This study, as another example of the role of miRNAs in lipid reprogramming, showed that miR-495-3p targets SPHK1 and, as a result, reprograms the sphingolipid pathway toward ceramide. Finally, these changes induce lethal mitophagy and suppress tumorigenesis in non-small cell lung cancer [55].

Some nonessential amino acids are essential to adapt cancer cells to high-pressured tumor niches. Glutamine (Gln), the most plentiful free amino acid in the vascular system in cooperation with glucose, is essential for fulfilling the anabolism requirement of proliferating cancer cells. Glutaminase (GLS) is the first enzyme in the TCA cycle that transforms glutamine to glutamate, which is then converted to α-ketoglutarate for metabolism. In mammals, two genes encode glutaminase, GLS and GLS2, which have distinct structures and are expressed in various regions. Glutamate is a precursor of glutathione (GSH), a significant cellular antioxidant that aids in the maintenance of proper immune responses. It has been found that the mitochondrial GLS protein is directly regulated through c-Myc activity. Indeed, two miRNAs, miR-23a and miR-23b, that target GLS expression are transcriptionally suppressed by c-Myc activity [56].

Another part of amino acid metabolism is related to one-carbon metabolism, which comprises a series of interlinking metabolic pathways, including the methionine and folate cycles. However, compared with our comprehensive understanding of the role of glycolysis and glutaminolysis in tumor cells, our knowledge of the contribution of one-carbon metabolism in cancer cell processes is in its infancy. Methionine adenosyltransferase (MAT) is a group of enzymes essential for *S*-adenosyl methionine (SAM) synthesis in all mammalian cells, especially hepatic cells [57]. Upregulation of miR-485-3p, miR-495, and miR-664 are correlated with low-level expression of MAT1A in HCC. On the other hand, inhibition of these miRNAs reduces cell growth and, conversely, promotes apoptosis. Higher nuclear expression of MAT1A is positively associated with reduced tumorigenicity, invasion, and metastasis [58].

As a result, cancer cells inhibit the expression of MAT1 and improve their invasion ability by increasing the mentioned miRNA expression. Stone et al. found a group critical to regulating miRNAs, including miR-125, miR-22, miR-488, miR-344-5p, and miR-484, that control several contributed enzymes to one-carbon metabolism [59]. As mentioned, glutamine metabolism has also been the target of recent studies to clarify the role of miRNAs in reprogramming cell metabolism in cancer cells and its role in drug resistance or sensitivity. Chang et al. showed that treating human malignant melanoma cells with temozolomide significantly reduced miR-203 expression. On the other hand, it became clear that miR-203 expression in temozolomide-resistant malignant melanoma cells is lower than in nonresistant cells. In addition, the levels of glutamine metabolism and glutaminase expression in these cells are higher.

The results showed that miR-203 leads to a decrease in its expression and an increase in the sensitivity of cells to temozolomide by targeting the 3′-UTR region of the glutaminase gene. Based on these results, it was suggested that miR-203 could be an antitumor agent by increasing the sensitivity of malignant melanoma cells to temozolomide by targeting glutaminase [60]. On the other hand, Liu et al. determined the role of miR-153 in regulating glutamine metabolism in glioblastoma. In general, miR-153 expression decreases in glioblastoma. Increasing its expression and the effect on glutamine regulation can be a therapeutic target in glioblastoma [61]. Also, Bacci et al. suggested that targeting amino acid metabolic reprogramming is effective in breast cancer cells. It was revealed that downregulation of the neutral and essential amino acid transporter SLC6A14 governed by enhanced miR-23b-3p expression impaired amino acid metabolism [62]. Other miRNAs and their mechanism in cancer cell metabolism reprogramming are summarized in Table 1.

**Table 1:** MiRNAs and their contribution to the metabolism reprogramming of cancer cells

MiRNAs	Oncogene/tumor suppressor (TS)	Cancer	Metabolic reprogramming	Mechanism	Refs.
miR-7	TS	Glioma	Repression of glucose metabolism	Targeting the 3′-UTR of IGF1-R	[16]
miR-143	TS	Lung and colon cancers	Repression of glucose metabolism	Inhibiting HK2	[29, 63]
miR-1291	TS	Renal cell carcinoma, breast and pancreatic cancer	Repression of glucose and lipid metabolisms	Targeting GLUT1, downregulating CPT1C expression	[19, 20]
miR-199a-5p	TS	Non-small cell lung cancer and liver cancer	Repression of the glucose metabolism	Downregulating GLUT 1 and inhibiting HK2 and glycolysis	[64, 65]
miR-199a-3p	TS	Renal cell carcinoma	Repression of the glucose metabolism	Inhibiting Glut1 and glycolysis	[26]
miR-138	TS	Renal cell carcinoma	Repression of the glucose metabolism	Inhibiting Glut1 and glycolysis	
miR-150	TS	Renal cell carcinoma	Repression of the glucose metabolism	Inhibiting Glut1 and glycolysis	
miR-532-5p	TS	Renal cell carcinoma	Repression of the glucose metabolism	Inhibiting Glut1 and glycolysis	
miR-33a/33b	TS	Hepatic cells	Repression of the glucose and lipid metabolisms	Inhibiting G6PC and PCK1 Inhibiting translation of CPT1A, CROT, and HADHB	[48, 66]
miR-122	Oncogene	Breast cancer HCC	Elevation of glucose metabolism Elevation of cholesterol metabolism	Targeting PKM2, Targeting hepatic fatty acid oxidation and inducing cholesterol synthesis	[31, 50]
Let-7a	TS	Glioma	Repression of glucose metabolism	Regulating PKM2 expression	[67]
let-7a-5p	TS	TNBC	Repression of glucose metabolism	Lowering the expression of GLUT12 and inhibiting aerobic glycolysis	[24]
miR-26a	Oncogene	Colorectal cancer cells	Elevation of glucose metabolism	Inhibiting the critical step of glycolysis entry into the TCA cycle by targeting PDHX	[33]
miR-375	TS	Gastric cancer Esophagus cancer	Repression of glucose metabolism	Targeting PDK1 and suppressing aerobic glycolysis	[68]
miR-23a/b 2009	TS	Prostate cancer cells	Repression of amino acid metabolism	Promoting glutamine metabolism by targeting glutaminase	[56]
miR-664, miR-485-3p, miR-495	Oncogene	Hepatocellular carcinoma	Repression of amino acid metabolism	Inhibiting MAT1A expression	[58]
miR-181a-5p	Oncogene	Liver cancer	Elevation of glucose metabolism	Reducing the level of MT-CYB and MT-CO2, upregulating HK2 and GLUT1, increasing LDH activity	[39]
miR-155 miR-210	Oncogene	Melanoma	Elevation of glucose metabolism	Increasing aerobic glycolysis and decreasing oxidative phosphorylation	[41]
miR-27a	TS	Colorectal cancer	Repression of glucose and lipid metabolism	Impairing oxidative phosphorylation, reducing TCA cycle and fatty acid β-oxidation through modulation of PGC-1α, CPT1A, ACAD9, citrate synthase, HK1 and HK2, DLAT, the E2 component of the PDH	[43]
miR-342-3p	TS	TNBC	Repression of glucose metabolism	Targeting MCT1 and reducing glycolysis	[45]
miR-146a-5p miR-155-5p	Oncogene	Renal cancer	Elevation of glucose and amino acid metabolisms	Targeting G6PD and TKT (PPP enzymes) Targeting SUCLG2 (TCA enzyme), targeting GATM (arginine metabolism enzyme)	[69]
miR-513c miR-3163	TS	Breast cancer	Repression of amino acid metabolism	Targeting glutamine metabolism, especially glutaminase	[70]

ORP8: oxysterol-binding protein-related protein 8; TS: tumor suppressor; UTR: untranslated region; IGF1-R: insulin-like growth factor 1 receptor; GLUT1: glucose transporter 1; CPT1A: carnitine palmitoyltransferase 1A; CPT1C: carnitine palmitoyltransferase 1C; CROT: carnitine octanoyltransferase; HADHB: hydroxyacyl-CoA dehydrogenase trifunctional multienzyme complex subunit beta; PKM2: enzyme pyruvate kinase M2; HCC: hepatocellular carcinoma; PDHX: pyruvate dehydrogenase complex component X; PDK1: pyruvate dehydrogenase kinase 1; MAT1A: methionine adenosyltransferase 1A; HK1: hexokinase 1; HK2: hexokinase 2; PGC-1α: peroxisome proliferator-activated receptor gamma coactivator 1-alpha; ACAD9: acyl-CoA dehydrogenase family member 9; E2: dihydrolipoyl acetyltransferase; PDH: pyruvate dehydrogenase; TKT: transketolase; G6PC: glucose-6-phosphatase catalytic subunit 1; mt-CYB: mitochondrial cytochrome B; mt-CO2: mitochondrial cytochrome C oxidase II; TNBC: triple-negative breast cancer; MCT1: monocarboxylate transporter 1; SUCLG2: succinate-CoA ligase GDP-forming subunit beta; GATM: guanidinoacetate *N*-methyltransferase; PPP: pentose phosphate pathway; TCA: tricarboxylic acid cycle; PCK1: phosphoenolpyruvate carboxykinase 1; PCK2: phosphoenolpyruvate carboxykinase 2; NSLC: non-small cell lung cancer

## Long noncoding RNAs regulate cancer metabolic reprogramming

The regulatory role of lncRNAs in cancer progression and their contribution to cancer cell metabolism reprogramming are two integrated and well-studied topics. Before the documented results based on the interaction of lncRNAs and metabolic enzymes, their role had been confirmed in controlling metabolism reprogramming [71]. For example, the activity of many lncRNAs as miRNA sponges can explicitly explain their role in the metabolism reprogramming of cancer cells. LncRNA H19 generally acts as an oncogene in various cancers, and its upregulation is correlated with tumor invasion and metastasis. In addition, recent evidence found that H19 expression is elevated in hypoxia stress and can target miR-let-7, decreasing glucose uptake [72]. Upregulation of lncRNA H19 is correlated with overexpression of PKM2, which promotes glycolysis and cell growth [73]. Furthermore, Sun et al. indicated that H19, in addition to let-7, increases lactate production by inhibiting miR-519D-3p and activating LDHA signaling [74].

The lncRNA urothelial carcinoma-associated 1 (UCA1) is another lncRNA that regulates glucose metabolism in cancer cells. UCA1, via the mTOR-STAT3/microRNA-143-HK2 axis, increases glycolysis in bladder cancer cells [75]. Hypoxia stress induces lincRNA-p21 to enhance glycolysis by disrupting the interaction between hypoxia-inducible factor-1α (HIF-1α) and von Hippel–Lindau tumor-suppressor protein (VHL). HIF-1α is a global transcriptional regulator of the hypoxic response degraded through VHL mediation. Therefore, lincRNA-21 leads to stabilization and accumulation of HIF-1α that promotes tumor progression and cancer cell survival under hypoxia conditions. Epigenetic regulation is an interesting mechanism of lncRNA regulatory function [76].

Furthermore, lncRNAs can regulate signaling pathways in cancer metabolic reprogramming at transcription, posttranscription, protein, and epigenetic levels. For instance, NF-κB interacting lncRNA (lncRNA NKILA) suppresses the NF-κB signaling pathway through direct interaction with the NF-κB/IκB complex. NKILA expression is declined in cancer metastasis such as breast cancer, and is associated with poor prognosis. Prostate cancer gene expression marker 1 (PCGEM1) can activate androgen receptors indicating its tumorigenic potential. PCGEM1 is overexpressed in prostate cancer and is pivotal in cancer metabolism regulation [77]. Hung et al. showed that PCGEM1 enhances glucose uptake by c-Myc activation. Glucose can subsequently be diverted to the pentose phosphate pathway, facilitating macromolecule production and requiring less effort to maintain redox equilibrium [78].

Zhu et al. also described another lncRNA that is overexpressed in response to nutrient deprivation and stimulates glucose reprogramming. Mechanistically, they identified binding motifs of HOXC-AS3 with SIRT6. HOXC-AS3 binding to SIRT6 hindered HIF1α contact inhibition, resulting in metabolic pathway reprogramming in breast cancer. Furthermore, they indicated that HOXC-AS3 could significantly suppress breast cancer progression, which suggests the potential of anti-HOXC-AS3 for breast cancer treatment [79]. Recently Li et al. investigated two essential aspects of the role of lncRNA in cancer cell reprogramming. Primarily, they surveyed the impact of exosomal lncRNAs derived from cancer-associated fibroblasts (CAFs), the interaction between vicinity cells, and the effect of their shared microenvironment on tumor progression. Secondly, they found the mechanism activity of lncRNA SNHG3 in the metabolism reprogramming of breast cancer cells. Pyruvate kinase M1/M2 (PKMs) catalyzes the last step of glycolysis and is often upregulated in different cancers. PKM is targeted by miR-330-5p, resulting in an decrease in glycolysis and reduced breast cancer cell proliferation. SNHG3, as a sponge for miR-330-5p, increases PKN expression and the glycolysis rate. Overexpression of SNHG3 reduced mitochondrial phosphorylation oxidative and, in contrast, promoted carboxylation glycolysis through the miR-330-5p/PKM pathway [80]. Luo et al. found that GAS6-AS1 inhibits tumor progression of lung adenocarcinoma in vivo and in vitro by negatively regulating GLUT1 expression. Usually, GAS6-AS1 is downregulated in cancers as a tumor suppressor associated with clinicopathological characteristics. They revealed that GAS6-AS1 suppressed GLUT1 expression through direct interaction with E2F1 (transcription factor), consequently reducing cell glycolysis. Disruption in mitochondrial function is a metabolic characteristic of tumor cells [81].

Regarding the pivotal roles of lncRNAs roles in molecular mechanisms in cancer progression, Zhao et al. investigated lncRNA MALAT1 activity in the mitochondria of HCC cells. They indicated the interaction of MALAT1 with various mitochondrial genes such as ND3, D-loop, CYTB, and COX2 by the RNA reverse transcription-associated trap sequencing (RAT-seq) technique [82]. RAT-seq enables the identification of the genome-wide targets for a specific lncRNA [83]. Furthermore, they found that MALAT1 knockdown leads to conspicuous changes in the CpG methylation of mtDNA and alterations in mitochondrial transcriptomes. These alterations were correlated with disruption in mitochondrial activities and structure, reduced ATP generation, and a low rate of oxidative phosphorylation. Mitochondrial metabolism reprogramming also was associated with alterations in epigenetic regulation and mitochondrial apoptosis in cancer cells [82].

Some recent studies have also considered the role of lncRNAs in cell reprogramming and their participation in tumor microenvironment regulation. For instance, Xu et al. found that through the extracellular exosome transmission of a myeloid-derived lncRNA called M2 macrophage polarization-associated lncRNA, tumor-associated macrophages (TAMs) promote aerobic glycolysis in HCC cells and their proliferation (lncMMPA). Regarding its mechanism, lncMMPA could polarize M2 macrophages and function as a sponge for miRNAs to interact with miR-548s and raise the mRNA level of ALDH1A3, which would then further encourage glucose metabolism and cell growth in HCC. Moreover, via interacting with miR-548 in vivo, lncMMPA boosted HCC cell proliferation. Clinically, lncMMPA expression is related to reduced patient survival from HCC and glycolysis in tumor-associated macrophages.

LncMMPA is crucial for controlling HCC malignancy and metabolic reprogramming of the miR-548s/ALDH1A3 pathway [84]. Also, Pan et al. revealed that lncRNA HCG18 has an oncogenic role, and its expression was increased in osteosarcoma. It was suggested that HCG18 has a critical role in regulating aerobic glycolysis by sponging miR-365a-3p to increase the expression of PGK1 in osteosarcoma cells [85].

In another study on colorectal cancer resistant to 5-fluorouracil (5-FU), it was clarified that the expression of lncRNA hCG11 increased and was associated with increased cell division, invasion, migration, glucose metabolism, and resistance to 5-FU. In addition, the results showed that lncRNA hCG11 inhibits the expression of miR-144-3P through sponging and siRNA network formation. On the other hand, miR-144-3P is a direct inhibitor of pyruvate dehydrogenase 4. It was verified through rescue studies that miR-144-3p inhibits glucose metabolism and 5-FU sensitization by targeting PDK4. Therefore, induced miR-144-3P expression overrode the function of HCG11 in cells resistant to 5-FU with high levels of lncRNA HCG11 and reversed 5-FU resistance by targeting PDK4 [86].

Recent investigations have also indicated the role of lncRNAs in fatty acid metabolism regulation during cancer progression. For instance, Liu et al. found that lncRNA ROPM was highly expressed in breast cancer stem cells (BCSCs). lncRNA RPOM can bind the 3′-UTR of PLA2616 and increase phospholipid metabolism, activating PI3K/AKT, Wnt/β-catenin, and Hippo/YAP signaling, which leads to the maintenance of BCSCs stemness [87]. Also, Wang et al. showed that MALAT1 (another type of lncRNA) plays a significant role in expressing AMP-activated protein kinase signaling genes in liver cancer cells. They showed that MALAT1 knockdown could reduce lipogenesis and lower cancer cell proliferation [88]. DNAJCs-AS1 is another lncRNA that is expressed in colorectal cancer, and inhibiting it can reduce the proliferation and metastatic behavior of CRC cells. This lncRNA can modulate the synthesis of fatty acids and the NF-κβ signaling pathway [89]. Logotheti et al. described the contribution of lncRNA SLC16A1-AS1 in induction metabolism reprogramming during bladder cancer progression. SLC16A1/MCT1 is a lactate transporter transcripted via prescription factor E2F1, which co-transcripts lncRNA SLC16A1-AS1 in conjunction with the SLC16A1/MCT1 sequence gene. Therefore, overexpression of SLC16A1-AS1 as a co-activator of the transcription factor leads to high-level expression of SLC16A1/MCT1 in turn. Consequently, this lactate transporter enhances mitochondrial respiration, glycolysis, and fatty acid β-oxidation (FAO) in bladder cancer cells [90]. LncRNA nuclear-enriched abundant transcript 1 (NEAT1) is an oncogene in multiple cancers. Liu et al. reported the role of lncRNA NEAT1 in regulating adipose triglyceride lipase (ATGL) expression and lipolysis disruption in HCC cells [91]. In contrast, it is reported that NEAT1 inhibition results in decreased HCC cell proliferation and upregulation of lipolysis rate through miR-124-3p overexpression. Interestingly, in different cancers, NEAT1 acts as an immune regulator [92]. NEAT1 is expressed at lower levels in tumor samples with high levels of cytotoxic CD8+ infiltration. Moreover, NEAT1 fosters tumor growth by reducing the expression of cyclic GMP–AMP synthase stimulator of interferon genes, which inhibits cytotoxic T cell-mediated immunity [93]. The crosstalk between the contribution of ncRNAs to cancer cell metabolic reprogramming and immune microenvironment remodeling has been thoroughly investigated in recent studies, which are well reviewed in ref. [94]. Moreover, numerous studies reported the interaction between lncRNAs such as PANDA, MEG3, RoR, and Wrap53, and staple factors of modulating hypoxia signaling pathways, which implies that lncRNAs may play a function in tumor metabolic regulation [95–97].

The role of lncRNAs in metabolism reprogramming in cancer cells is not restricted to glycolysis. For instance, lncRNA CCAT2 (colon cancer-associated transcript 2) promotes glycolysis and plays a pivotal role in increased glutamine metabolism in various cancers [98]. A recent example of the participation of lncRNAs in glutamine metabolism is lncRNA HOX transcript antisense intergenic RNA (HOTAIR), which significantly increases in different cancers. HOTAIR acts as a sponge for miR-126-5p, leading to increased glutamine metabolism in glioma. MiR-126-5p directly targets GLS mRNA and reduces its expression in RNA and protein levels. Hence, HOTAIR can promote glutamine metabolism through the miR-126-5p-GLS axis, which causes tumor progression in glioma [99]. Aspartate metabolism is confirmed to be altered to support malignant activities in cancer cells. According to recent evidence, argininosuccinate synthase 1 (ASS1) is a crucial enzyme for limiting aspartate metabolism and has a reduced expression level as a tumor suppressor in cancer cells [100]. Chen et al. reported the role of LINC01234 in aspartate metabolism. LINC01234 is overexpressed and correlated with a high tumor progression and migration rate, poor prognosis, and drug resistance in HCC patients. Chen’s team realized that LINC01234 could suppress ASS1 expression by binding to its promoter and inhibiting transcription. They showed that LINC01234, by alteration in aspartate metabolism, could be a potential therapeutic target in HCC [100]. Table 2 summarizes lncRNAs and their contribution to metabolism reprogramming in various cancer cells.

**Table 2:** LncRNAs and their contribution to the metabolism reprogramming of cancer cells

lncRNA	Oncogene/TS	Cancer	Metabolic reprogramming	Mechanism	Refs.
lincRNA-p21	TS	Prostate cancer	Repression of glucose metabolism	Inhibiting PKM2 expression, reducing pyruvate production, and inhibiting cell proliferation	[101]
lncRNA H19	TS	Ovarian cancer	Elevation of glucose metabolism	Enhancing glucose consumption, lactate production, and PKM2 expression by sponging miR-324-5p	[102]
TP53TG1	Oncogenic	Brain tumor cells	Elevation of glucose metabolism	Promotes the expression of glucose metabolism-related genes LDHA and IDH1	[103]
MAFG-AS1	Oncogenic	Colorectal Ccncer	Elevation of glucose metabolism	Sponging miR-147b and activation of NDUFA4, causing an upregulation of PDK1, PFK1, and PKM2	[104]
FEZF1-AS1	Oncogenic	Colorectal cancer	Elevation of glucose metabolism	Binds and increases the stability of PKM2	[105]
BCYRN1	Oncogenic	Non-small cell lung cancer	Elevating glucose metabolism	Increasing the expression levels of PKM2 and inducing glycolysis via the miR-149/PKM2 axis	[106]
AC020978	Oncogenic	Non-small cell lung cancer	Elevation of glucose metabolism	Promotion of glycolysis by direct interaction with PKM2 and enhancing PKM2 protein stability Promotes the nuclear translocation of PKM2 and regulate PKM2-enhanced HIF-1α transcription activity	[107]
LINC00689	Oncogenic	Glioma	Elevation of glucose metabolism	Promoting glycolysis by sponging of miR-338-3p and increasing PKM2 expression	[108]
LINC01554	TS	Liver cancer	Repression of glucose metabolism	Decreasing in PKM2 expression and reduced glycolysis and cancer cell progression	[109]
SOX2OT	Oncogenic	Liver cancer	Elevation of glucose metabolism	Promoting glycolysis by sponging miR-122-5p and activating PKM2	[110]
NEAT1	Oncogenic	HCC	Elevation of lipid metabolism	Upregulating adipose triglyceride lipase (ATGL) expression and increasing lipolysis	[91]
lincRNA DYNLRB2-2	Oncogenic	–	Elevation of lipid metabolism	Upregulating ABCA1 and increasing cholesterol metabolism	[111]
lncRNA HULC	Oncogenic	HCC	Elevation of lipid metabolism	Overexpression of sphingosine kinase 1, Increasing the synthesis of sphingomyelin	[112]
HOTAIR	Oncogenic	HCC	Elevation of amino acid metabolism	Upregulation in glutamine metabolism by sponging miR-126-5p to regulate glutaminase	[113]
CCAT2	Oncogenic	Variety of cancers	Elevation of amino acid metabolism	Upregulation in glutamine metabolism by the expression of an alternative splicing isoform of GLS (glutaminase isoform C)	[114]
PCGEM1	Oncogenic	Prostate cancer	Elevation of amino acid metabolism	Activating c-Myc, regulating PPP, glutamine, and TCA pathways	[78]

SPHK1: sphingosine kinase 1; TS: tumor suppressor; GAC: glutaminase isoform C; PCGEM1: prostate cancer gene expression marker 1; PKM2: enzyme pyruvate kinase M2; LDHA: lactate dehydrogenase A; IDH1: isocitrate dehydrogenase 1; NDUFA4: NADH dehydrogenase (ubiquinone) 1 alpha subcomplex 4; PDK1: pyruvate dehydrogenase kinase 1; PFK1: phosphofructokinase-1; HIF-1α: hypoxia-inducible factor 1-alpha; ABCA1: ATP binding cassette subfamily A member 1; HCC: hepatocellular carcinoma; GLS: glutaminase isoform C; PPP: pentose phosphate pathway; TCA: tricarboxylic acid cycle

## CircRNAs regulate cancer metabolic reprogramming

Moreover, circRNAs, like lncRNAs, can act as miRNA sponges and profoundly alter metabolic and biological processes involved in tumor progression. Interestingly, some circRNAs regulate metabolism routes such as glycolysis, glutamine metabolism, and the expression of key enzymes in metabolic pathways [115]. A recent study highlighted the role of nuclear genome circRNAs in regulating mitochondria function during oxidative phosphorylation. Gong et al. indicated that the expression of circ-PUM1, localized in mitochondria, is associated with HIF1α accumulation under hypoxia conditions in esophageal squamous cell carcinoma cell lines. They found that circ-PUM1 is a scaffold for UQCRC1 and UQCRC2, two essential subunits of complex III in the respiratory chain.

Furthermore, circ-PUM1 silencing is correlated with a decrease in oxidative phosphorylation, intracellular oxygen concentration, and mitochondrial membrane potential. They also showed that circ-PUM1 downregulation disrupts complex III functions and leads to cleavage of caspase3 in esophageal squamous cell carcinoma cell lines [116]. A novel and the interesting elaborated study was conducted by Liu et al. regarding the cancer stem cells concept concerning circRNAs contribution to cancer cell reprogramming. Cancer stem cells are an unsolved barrier in cancer therapy. Recently, various studies indicated that cancer cells could adapt to adverse environmental factors and different chemical drugs mediated by cancer stem cells. Conversely, exosomes are extracellular vesicles that act as cell communications vehicles. Liu et al. found that exosomes containing circ-CARM1 derived from breast cancer stem cells can regulate glycolysis in breast cancer cells. Circ-CARM1 sponges miR-1252-5p, which directly targets 6-phosphofructo-2-kinase/fructose-2,6-biphosphatase 2 (PFKFB2) mRNA [117].

Overexpression of circMAT2B is correlated with poor prognosis in HCC. Furthermore, circMAT2B upregulation in hypoxia accelerates glycolysis by regulating the miR-338-3p/PKM2 axis. CircFOXM1 has a similar effect on glycolysis and PKM2 regulation in melanoma cells. In addition, circRPN2 suppressed HCC aerobic glycolysis and metastasis by accelerating enolase 1 (ENO1) degradation and regulating the miR-183-5p/FOXO1 axis [118].

It has also been suggested that miR-143-3p inhibited glycolysis and lactate production. Hence, targeting miR-143-3p by circFOXM1 can lead to high PKM2 activity and upregulated glycolysis. Circ-NRIP1 is another oncogenic circRNA overexpressed in gastric cancer [119]. Liu et al. indicated that circ-NRIP1 knockdown significantly reduced proliferation, migration, PKM2 expression, and glycolysis. It was proposed that these anticancer effects were caused, at least in part, by the competitive targeting of miR-186-5p [120]. In gallbladder cancer cells, circFOXP1 sponges miR-370 and upregulates PKLR expression, subsequently accelerating the Warburg effect [121]. In a study on complicated colon cancer models, exosome-delivered circular RNA hsa_circ_0005963 (ciRS-122) was delivered to chemosensitive cells and led to increased expression of PKM2 and glycolysis via miR-122 inhibition. In contrast, ciRS-122 suppression reversed chemoresistance and reduced glycolysis in colorectal cancer cells [122].

The study by Huang et al. showed the role of circKIF4A in reprogramming glucose metabolism in breast cancer distant metastasis. Based on the findings of the liver, breast cancer, and cell line metastatic tissue, it was clarified that circKIF4A plays a role in cell migration, glucose absorption, and lactate production. Further research revealed that circKIF4A might sponge miR-335, which influenced the expression of the ALDOA/OCT4 protein and controlled the expression of HK2/PKM2. The circKIF4A-miR-335-OCT4/ALDOA-HK2/PKM2 axis was shown in this work to be essential for the metabolic reprogramming of breast cancer, suggesting that this axis may represent a potential therapeutic target for the treatment of liver metastases of breast cancer [123]. Li et al. found that circDNMT1 promotes the malignancy of gastric cancer by inhibiting miR-576-3p and, as a result, increases the expression of HIF-alpha. They showed that circDNMT1 as an oncogenic ncRNA is regulated in gastric cancer cells and tissues, and is related to shorter survival of patients and pathological T stages. CircDNMT1, as a sponge, inhibits miR-576-3p, which negatively regulates HIF-alpha. These findings show that it is possible to inhibit the invasion and division of migration and glycolysis in gastric cancer cells by knocking down circDNMT1 as a new therapeutic target [124]. A valuable study that shows the importance of circRNAs as new therapeutic targets is that by Qu et al. [125]. This study used propofol as an inhibitor of malignancy in ovarian tumors. Molecular results showed that propofol leads to the inhibition of circular RNA-zinc finger RNA binding protein (circ-ZFR). Circ-ZFR mechanistically leads to the inhibition of superoxide dismutase and glycolysis. Therefore, its expression is associated with inhibiting the expression of miR-212-5P and increasing the expression of superoxide dismutase, which ultimately leads to an increase in cell division, invasion, migration, and glycolysis in cancer cells. Therefore, propofol is an antitumor therapy targeting circ-ZFR [125].

The pentose phosphate pathway, as a parallel metabolic pathway with glycolysis, also plays a vital role in meeting the needs of cancer cells. Chen et al. showed the role of circ-0003215 in suppressing the pentose phosphate pathway and colorectal cancer malignancy. In general, the expression of circ-0003215 in colorectal cancer cells is low, and it is inversely associated with the tumor size, the TNM stage, and lymph node metastasis. In addition, the decrease in the expression of circ-0003215 is the result of RNA degradation through the m6A reader protein YTHDF2. The experiments showed that circ-0003215 inhibits migration, invasion, cell division, and metastasis of colorectal tumors. Circ-0003215 acts as a sponge for miR-663B, activating its DLG4 target. Through the K48-linked ubiquitination of glucose-6-phosphate dehydrogenase (G6PD), DLG4 hindered the pentose phosphate pathway. We have discovered circ 0003215 with the m6A modification to be a novel metabolic glucose reprogramming regulator that inhibited the pentose phosphate pathway and the malignant phenotype of colorectal cancer by acting on the miR-663b/DLG4/G6PD axis [126].

Since lipids play massive roles in energy storage, the structure of cells, signaling, and messengers, changes in lipid metabolism can lead to inevitable damage in cells. Lipid metabolism in cancer cells experience various alternations; therefore, lack of structural lipids for constructing cell membranes, disruption in cell signaling, and eventually, cell growth happens in these kinds of cells [127, 128].

CircRNAs can influence lipid metabolism in cancer cells [129]. The circRNA, CUT-like homeobox 1 (CUX1), encodes a 113 amino acid protein (p113), which interacts with Zuotin-related factor 1 (ZRF1) and results in higher production of fatty acids, mitochondrial complex I activity, and therefore, aggressiveness and tumorigenesis of neuroblastoma (NB) [130]. Circ-MBOAT2 levels up in cholangiocarcinoma (ICC) cells since it can combine with PTBP1 and protect it from degradation through ubiquitin/proteasome. This results in the facilitation of lipid reprogramming in ICC cells [131]. Wu et al. claimed that circRIC8B plays a crucial role in lipid accumulation and increased proliferation through the miR-199b-5p/LPL axis in chronic lymphocytic leukemia (CLL) cells [132].

Recent research has focused on the role of circRNAs in the metabolism of amino acids, particularly glutamine. Two essential amino acid transporter proteins, solute carrier family A1 member 5 (SLC1A5) and solute carrier family A7 member 5 (SLC7A5), are overexpressed in lung cancer. Furthermore, the metabolic reprogramming of cancer cells is directly regulated by a mitochondrial variant of SLC1A5. SLC1A5 and SLC7A5 have been proposed as critical therapeutic targets in cancer metabolism due to their significance in tumor metabolism [133]. According to Xue et al., circ-LDLRAD3 and miR-137 are contradictorily expressed in non-small cell lung cancer cell lines and tissues, so overexpression of circ-LDLRAD3 is correlated with downregulation of miR-137 and clinicopathological properties. They found that circ-LDLRAD3 controls SLC1A5 via sponging miR-137 in non-small cell lung cancer cells, controlling tumor cell apoptosis, migration, and proliferation [133]. Also, Ma et al. revealed that circ_0025033 could regulate SLC1A5 expression in ovarian cancer cells via sponging hsa_miR-370-3p. They suggested that circ_0025033 promotes ovarian cancer cell malignant behaviors and glutamine metabolism via the hsa_miR-370-3p/SLC1A5 axis [134].

Glutamate oxaloacetate transaminase 1 (GOT1) and 2 (GOT2) generate α-ketoglutarate and regulate glutamate metabolism by indirectly participating in the TCA cycle. Zhu et al. indicated the association of hsa_circRNA_103809 overexpression and cisplatin-resistance in non-small cell lung cancer cells. Mechanistically, hsa_circRNA_103809 targets miR-377-3p expression and indirectly upregulates GOT1 expression, resulting in cisplatin resistance and lung tumor progression [135]. Additionally, circ-0003028 was shown to be significantly elevated in non-small cell lung cancer tissues and cell lines, and its expression level was strongly connected with aggressive biological features such as colony formation and invasion in non-small cell lung cancer cells. Circ-0003028 binds to miR-1298-5p directly, and GOT2 is a direct target of miR-1298-5p. The circ-0003028/miR 1298-5p/GOT2 axis, taken combined, might represent an important therapeutic target for non-small cell lung cancer [136]. The body of research provided thus far has indicated that the different reported circRNAs may operate as cancer promoters in various malignant forms by sponging miRNAs and modulating PKM2 expression.

Serine and glycine (SG) metabolism is an essential component of amino acid metabolism that can promote cell growth. Liu et al. claimed that in a p53-dependent manner, the overexpression of circMYH9 modulates serine/glycine metabolism and redox homeostasis to encourage the development of colorectal cancer [137].

Other types of noncoding RNA, including piRNA, may be implicated in mediating metabolic reprogramming in addition to circRNAs. However, information is scarce on the subject. Nonetheless, the increasingly recognized activities of piRNAs, which imply their ability to control numerous facets of cancer, require further study [138]. The role of circRNAs and their mechanism in cancer cell reprogramming are presented in Table 3.

**Table 3:** CircRNAs and their contribution to the metabolism reprogramming of cancer cells

circRNAs	Oncogene/TS	Cancer	Metabolic reprogramming	Mechanism	Refs
circMYLK	Oncogene	Lung cancer	Elevating glucose metabolism	Overexpression of GLUT3 by targeting miR-195-5p	[139]
circMAGI3	Oncogene	Lung cancer	Elevation of glucose metabolism	Overexpression of HDGF by targeting miR-515-5p	[140]
circAGFG1	Oncogene	Lung cancer	Elevation of glucose metabolism	Overexpression of HIF-1*α*	[141]
circ-ENO1	Oncogene	Lung cancer	Elevation of glucose metabolism	Regulating ENO1 expression by targeting miR-22-3p	[142]
circRAD18	Oncogene	Papillary thyroid cancer	Elevation of glucose metabolism	Upregulation of PDK1 by targeting miR-516b	[143]
circRHBDD1	Oncogene	HCC	Elevation of glucose metabolism	Through an m6A dependent manner	[144]
circCARM1	Oncogene	Breast cancer	Elevation of glucose metabolism	Upregulating PFKFB2 by sponging miR-1252-5p	[117]
piR-Hep1	Oncogene	HCC	Elevation of glucose metabolism	Activating glycolysis through PI3K/Akt signaling pathway	[145]
snoRD113-1	Oncogene	HCC	Elevation of glucose metabolism	Through PI3K/Akt signaling pathway	[146]
hsa_circ_0018180 (circPARD3)	Oncogenic	Head and neck squamous cell carcinoma	Elevation of glucose metabolism	Through the miR-5194/ENO1 axis	[147]
circMBOAT2	Oncogene	ICC	Elevation of lipid metabolism	Stabilizing PTBP1 to facilitate FASN mRNA cytoplasmic export	[148]
circRIC8B	Oncogene	CLL	Elevation of lipid metabolism	Acting as a sponge of miR-199b-5p and preventing it from decreasing the level of lipoprotein lipase mRNA	[132]
circ-LDLRAD3	Oncogene	Lung cancer	Elevation of amino acid metabolism	promoting glutamine metabolism through upregulation of SLC1A5 by targeting miR-137	[133]
circ-103809	Oncogene	NSCLC	Elevation of amino acid metabolism	Upregulating GOT1 and promoting glutamine metabolism by targeting miR-377-3p	[149]
circ-0003028	Oncogene	Lung cancer	Elevation of amino acid metabolism	Overexpression of GOT2 by miR-1298-5p	[136]
circMYH9	Oncogene	CRC	Elevation of amino acid metabolism	Promotes the expression of serine and glycine synthesis enzymes to facilitate serine and glycine biosynthesis though p53-mediated upregulation of PHGDH	[137]
circ_0025033	Oncogene	Ovarian cancer	Elevation of amino acid metabolism	Promotes ovarian cancer cell malignant behaviors and glutamine metabolism via the hsa_miR-370-3p/SLC1A5 axis	[134]

GLUT3: glucose transporter 3; TS: tumor Suppressor; HDGF: heparin-binding growth factor; HIF-1α: hypoxia-inducible factor 1-alpha; ENO1: enolase 1; PDK1: pyruvate dehydrogenase kinase 1; HCC: hepatocellular carcinoma; ICC: intrahepatic cholangiocarcinoma; CLL: chronic lymphocytic leukemia; PFKFB2: 6-phosphofructo-2-kinase/fructose-2,6-biphosphatase 2; PI3K/Akt: phosphoinositide-3-kinase/protein kinase B (PKB, or Akt); PTBP1: polypyrimidine tract-binding protein 1; FASN: fatty acid synthase; SLC1A5: solute carrier family 1 member 5; GOT1: glutamic-oxaloacetic transaminase 1; GOT2: glutamic-oxaloacetic transaminase 2; NSCLC: non-small cell lung cancer; CRC: colorectal cancer; PHGDH: phosphoglycerate dehydrogenase

## Targeting ncRNAs as a therapeutic approach in cancer treatment

According to the prominent roles of ncRNAs in the development of cancer progression and their functions in affecting hallmarks of almost all malignancies [150–154], and also regarding their association with the immune system [155] and neurological [156] disorders, numerous ncRNAs have been targeted via multiple methods to dampen their oncogenic roles [157]. As discussed, ncRNAs could act as a tumor oncogene or suppressor agent. Generally, disrupting the network of oncogene ncRNAs has been performed in two significant arms as follows.

First, cancers mainly show a reduction of tumor suppressor ncRNAs. Thus, a strategy would be importing an exogenous ncRNA such as miRNA (called miRNA mimics) to restore the lost endogenous antitumor miRNA, possibly through designing a viral vector with the ability to express particular miRNAs [158]. For instance, it was reported that miR-15a and miR-16-1 are reduced in leukemia, particularly CLL [159]; thus, upregulation or inserting miR-15a and miR-16-1 mimics as well as vectors encoding miR-15a and miR-16-1 could enhance anti-apoptotic protein expression [160]. Secondly, direct inhibition of ncRNAs by constructing reverse complementary strains such as antisense oligonucleotides (ASO) and miRNA sponges that target oncogenic ncRNAs [161, 162]. The miRNA sponges are a novel antitumor therapy that can suppress the function of several oncogenic miRNAs at once through an artificial interfering lncRNA that possesses multiple binding sites for many miRNAs [163, 164]. A small interfering RNA (siRNA) is a double-stranded RNA homologous to the ncRNAs, which were incorporated into a multiprotein RNA-induced silencing complex with the ability to induce endonucleolytic cleavage of target ncRNA [165].

Taken together, various RNA-based therapeutic approaches have been developed, such as miRNA mimics, ASO, miRNA sponges, short hairpin RNAs (shRNA), siRNA, therapeutic circular RNAs (circRNA), ASO antimicroRNAs (antimiR), and also gene editing using the CRISPR–Cas9 system [157, 160]. Among these approaches, 11 ASOs and siRNAs that could target the pre-mRNA splicing and induce gene downregulation attained the approval of the United States Food and Drug Administration (FDA) and European Medicines Agency (EMA); however, some are utilized in cancer cases [157].

### Targeting miRNA

Although various types of ncRNAs are associated with cancer, the most studied one is miRNA, accounting for about 40% of cancer publications [166]. Based on how miRNAs could facilitate cancer development, therapeutic strategies are divided into restoring and blocking miRNAs.

#### Restoring the lost endogenous miRNAs

One of the mechanisms that results in downregulating tumor suppressor miRNA is epigenetic silencing. Some small molecules, such as hypomethylating factors, could restore the miRNA reserves. In this regard, 5-azacytidine and decitabine are two agents with a hypomethylating capacity approved and evaluated in cases with myelodysplastic syndromes (MDS) [167]. It was shown that miRNA-124a was inactivated in several cancers by the hypermethylation process in CpG island, and 5-azacytidine or decitabine promoted the expression of some ncRNAs, including miRNA-124a [168]. Interestingly, a recent study demonstrated that the progression-free survival (PFS) of patients with MDS was significantly better in groups receiving decitabine than 5-azacytidine. Similarly, decitabine induced 67.2% overall response rates (ORRs), while 5-azacytidine showed 44% [169]. Another small molecule is enoxacin, which has antibacterial activities and could boost the expression of miRNAs by interacting with TARBP2, a miRNA biosynthesis protein [170]. Enoxacin induced an antiproliferative effect on prostate tumor (PC-3)-bearing mice, possibly by elevating the activities of caspase-3/9 and releasing cytochrome-c. Besides, enoxacin reduced the expression of anti-apoptotic agents such as Bcl-2 and MCL-1 [171].

Another method is the application of miRNA mimics, which has been recommended to be delivered via a confident delivery strategy to enhance their stability and uptake [172]. Accordingly, in the murine orthotopic NB model, the targeted delivery of miR-34a using a nanoparticle (NP) coated with neuroblastoma-specific antibodies suppresses the growth of tumor cells [173]. In KRAS-activated non-small cell lung cancer (NSCLC)-bearing mice, local delivery of miR-34a and let-7 mimics through neutral lipid emulsions decreased the tumor burden significantly [174]. A recent phase I trial investigated a liposomal miR-34a mimic in patients with refractory solid tumors [most were HCC, melanoma, renal cell carcinoma (RCC), and lung cancer]. Liposomal miR-34a mimic was administered to 85 patients following dexamethasone premedication and resulted in frequently observed grade 3/4 laboratory abnormalities and manageable adverse events (AEs); however, with no complete responses (CR), and only 4 out of 85 patients showed partial responses (PR) [175].

Engineered vectors could allow specific miRNAs to be expressed in the targeted microenvironment [160]. This strategy was conducted in primary human HCC, where miR-26a reduced the cancer situation while its re-expression exhibited antitumor functions [176]. In this way, one study designed a vector system, an adeno-associated virus, and utilized it to administer miR-26a into an HCC mouse model, which consequently led to the suppression of tumor proliferation and induction of apoptosis in tumor cells [177].

#### Blocking of oncogenic miRNAs

Chief methods for blocking the oncogenic miRNAs are based on ASOs [160]. Locked nucleic acids (LNAs) are ASOs with the strongest affinity. Modifying oligonucleotides with a methylene bridge that could link the 2′-O atom and the 4′-C atom, ASOs provide a perfect conformation for Watson–Crick binding to the oncogenic miRNA [178]. There are numerous LNA-based ASO strategies applied in preclinical studies. Accordingly, miR-380-5p was shown to be highly expressed in mouse embryonic stem cells and neuroblastomas, and has oncogenic roles via inhibition of p53 expression through binding a conserved sequence in the 3′ untranslated region (UTR) of p53. The application of antagonist LNA-anti-miR-380-5p decreased the size of tumors in orthotopic NB mice, resulting in p53 regulation [179]. Short-length LNA ASOs with up to eight nucleotides also target the 5′ end of miRNAs [180]. The advantage of tiny LNA ASOs is observed when multiple miRNAs are the targets. Therefore, a more extended sequence is required when targeting a specific miRNA [160]. Indeed, a challenge to applying tiny LNA ASOs is the off-target activation of the therapeutic agent [180]. In this regard, an anti-miR-155 tiny LNA showed a suppressive effect against CLL and Waldenstrom macroglobulinemia by inhibiting the proliferation of tumors in vitro and also reducing the number of leukemic cells in vivo [181].

To broaden the range of targeted miRNAs, a new RNA-based therapy called miRNA sponges was developed to contain multiple complementary sequences and target multiple miRNAs [161]. It was reported that miRNA sponges with the ability to target the miR-17–92 cluster, miR-17, miR-18a, miR-19, and miR-92a, successfully silenced each miRNA of the cluster simultaneously. Besides, the miRNA sponges targeting the miR-17–92 cluster showed a more substantial capacity to inhibit the proliferation of B-cell lymphoma cells in vitro compared with ASOs with a single miRNA target [182]. It was demonstrated that miR-19 and miR-155 could reverse the tumor suppression process by affecting suppressor of cytokine signaling-1 (SOCS1) and its downstream effector p53. In a study of human myeloma cells and mouse leukemia cells, miRNA sponges against miR-19 and miR-155 silenced these miRNAs leading to the induction of SOCS1 and p53 in tumor cells [183].

### Targeting lncRNAs

Based on the previous investigations, miRNAs were the first ncRNAs as candidates for targeting by RNA-based therapeutic approaches [184], contrary to lncRNAs, whose studies were limited [185]; however, this context will be discussed further. RNA interference (RNAi) could be activated via cellular events, and as a result, two chief RNAs, i.e., miRNAs and siRNAs, participate in multiple cellular pathways [186, 187], implying the pivotal role of siRNAs similar to miRNAs. Several siRNAs are evaluated in knocking down lncRNAs in cell lines [188]; however, possibly due to the challenges of drug delivery methods and the low bioavailability of siRNAs in living bodies, in vivo studies of siRNAs have been difficult to perform [189]. Accordingly, a siRNA able to target MALAT1, a lncRNA that was previously demonstrated to have roles in NSCLC metastasis and acts as an oncogene lncRNA [190], was shown to suppress the growth of prostate cancer cell lines as well as their invasion and migration, and also promote cell cycle arrest [191]. It has been demonstrated that HOTAIR acts as an oncogene lncRNA in several tumors such as lung, pancreas, colorectal, liver, and breast [185, 192]. In an in vitro study, the invasion of breast tumors was suppressed after siRNA treatment due to HOTAIR knockdown [192]. In a study of colon cancer, a siRNA targeting NEAT1, an oncogene lncRNA, which was encapsulated into a chitosan NP system, showed antitumor activities by inhibiting the growth and metastasis of colon cancer cell lines (HCT116, LoVo, and SW480) possibly via NEAT1 knockdown and inhibiting miR-377-3p as a result [193].

In addition to siRNAs, ASOs could also be used as a therapeutic strategy to target lncRNAs [185]. The development of LNA and S-constrained ethyl (cEt) modifications have been considered the major advances in the chemistry of ASOs, with the ability to improve their pharmacokinetic features [194, 195]. The subcutaneous administration of cEt ASO against MALAT1 in luminal B breast cancer-bearing mice reduced the metastasis of tumors by 80% compared with nontreated models [196]. Targeting MALAT1 by ASO was also evaluated in a study of lung cancer, where this treatment reduced the metastasis of lung tumors (A549 cell line), implying the capacity of MALAT1 as a potent therapeutic target as well as a predictive marker [197].

Recent advancements in genome editing methods such as CRISPR–Cas9 provide a novel therapeutic platform for silencing lncRNAs [198, 199]. In this regard, dead-Cas9 is linked to transcriptional inhibitors and guides RNAs to direct this fusion protein to the targets to induce silencing [200]. Interestingly, guide RNAs could target the promoters of more than 16,000 lncRNAs in the human genome [201]. Therefore, the CRISPR-based method could be a potential novel strategy in the induction of transcriptional silencing of lncRNAs in future cancer studies; however, there is a long way to go to take advantage of CRISPR methods at the clinical levels, similar to preclinical ones [196]. Another circRNA that siRNAs have silenced, and for which the effect has been seen in the metabolism of cancer cells, is circNFATC3. The knockdown of circNFATC3 can regulate oxidative phosphorylation and the TCA cycle, directly affecting mitochondrial function and maintaining a dormant metabolic phenotype with low respiratory capacity and glycolysis [202].

### Targeting circRNAs

CircRNAs have the potential to be effective therapeutic targets because they are highly stable and typically express themselves in a tissue- or cell-type-specific manner. Several approaches to targeting circRNAs for medicinal purposes have been developed. These include conditional circRNA knockdown mediated by a cre-dependent shRNA, which is then processed into siRNA to induce circRNA cleavage and CRISPR–Cas9-mediated circRNA knockout via removal of the intronic complementary sequence flanking circularized exon involved in circRNA biogenesis. CircRNAs are directly targeted by CRISPR–Cas13-mediated circRNA knockdown to cause circRNA cleavage, circRNA overexpression is caused by the circRNA expression plasmid, and circRNA cleavage is brought on by siRNA/shRNA that target the back-splice junction of circRNAs [203]. The oncogene circPITX1 was highly expressed in gliomas. It has been shown that the knockdown of circPITX1 by siRNA (si-circPITX1) inhibits glucose consumption, lactate production, and the ATP level [204].

## Conclusions

Dysregulated ncRNAs, particularly miRNAs, lncRNAs, and circRNAs, have been identified as significant participants in the metabolic reprogramming of cancer cells by regulating individual genes and modulating key molecular processes, including the glycolytic function, lipid anabolic and catabolic reactions, and amino acid metabolism. MiRNAs appear to be the most extensively and intensively studied ncRNAs in many cancers, with promising results in preclinical studies. More significantly, recognizing the precise mechanism of ncRNAs on metabolic reprogramming of cancer cells will provide a robust theoretical foundation for future precision medicine. Furthermore, only a few of these ncRNAs are stable in body fluid, allowing for a noninvasive liquid biopsy approach. More research is needed to identify additional circulating ncRNAs for easy clinical diagnosis. Altogether, due to their diverse interactions and connections with critical cellular pathways frequently dysregulated in cancer, metabolism-associated ncRNAs may offer a novel approach to the early detection and personalized treatment of a wide range of cancers.

## Data Availability

Not applicable.

## Abbreviations

CSCs: Cancer stem cells
piRNA: PIWI-interacting RNA
snoRNAs: Small nucleolar RNAs
ROS: Reactive oxygen species
PPP: Pentose phosphate pathway
TCA: Tricarboxylic acid cycle
FA: Fatty acid
GLUT: Glucose transporter
TNBC: Triple-negative breast cancer
HK2: Hexokinase 2
PDK: Pyruvate dehydrogenase complex
mt-CYB: Mitochondrial cytochrome B
mt-CO2: Mitochondrial cytochrome C oxidase II
HCC: Hepatocellular carcinoma
mitomiRs: Mitochondrial miRNAs
PCK2: Phosphoenolpyruvate carboxykinase 2
HMEX: Human melanoma-derived exosomes
HADF: Human adult dermal fibroblasts
PDK1: Pyruvate dehydrogenase kinase isozyme 1
MCT1: Monocarboxylate transporter 1
CAFs: Cancer-associated fibroblasts
CPT1A: Carnitine palmitoyltransferase 1A
PPARδ: Peroxisome proliferator-activated receptor δ
HMGCS1: 3-Hydroxy-3-methylglutaryl-CoA synthase 1
DHCR7: 7-Dehydrocholesterol reductase
HMGCR: 3-Hydroxy-3-methylglutaryl-CoA reductase
SQLE: Squalene epoxidase
ACSL/SCD: Acyl-CoA synthetase/stearoyl-CoA desaturase
PPARα: Peroxisome proliferator-activated receptor α
PPARGC1B: Peroxisome proliferator-activated receptor gamma coactivator 1-beta
S1P: Sphingosine-1-phosphate
SPHK1: Sphingosine kinase 1
LDHA: Lactate dehydrogenase A
Gln: Glutamine
GLS: Glutaminase
GSH: Glutathione
MAT: Methionine adenosyltransferase
SAM: *S*-adenosyl methionine
UCA1: Urothelial carcinoma-associated 1
HIF-1α: Hypoxia-inducible factor-1α
VHL: Von Hippel–Lindau tumor-suppressor protein
PCGEM1: Prostate cancer gene expression marker 1
PKMs: Pyruvate kinase M1/M2
TAMs: Tumor-associated macrophages
5-FU: 5-Fluorouracil
BCSCs: Breast cancer stem cells
FAO: Fatty acid β-oxidation
NEAT1: Nuclear-enriched abundant transcript 1
ATGL: Adipose triglyceride lipase
CCAT2: Colon cancer-associated transcript 2
HOTAIR: HOX transcript antisense intergenic RNA
ASS1: Argininosuccinate synthase 1
PFKFB2: 6-Phosphofructo-2-kinase/fructose-2,6-biphosphatase 2
ENO1: Enolase 1
G6PD: Glucose-6-phosphate dehydrogenase
CUX1: CUT-like homeobox 1
ZRF1: Zuotin-related factor 1
NB: Neuroblastoma
ICC: Cholangiocarcinoma
CLL: Chronic lymphocytic leukemia
SLC1A5: Solute carrier family A1 member 5
SLC7A5: Solute carrier family A7 member 5
GOT1: Glutamate oxaloacetate transaminase 1
SG: Serine and glycine
ASO: Antisense oligonucleotides
siRNA: Small interfering RNAs
shRNA: Short hairpin RNAs
circRNA: Circular RNAs
antimiR: Anti-microRNAs
FDA: Food and drug administration
EMA: European medicines agency
MDS: Myelodysplastic syndromes
PFS: Progression-free survival
ORRs: Overall response rates
NP: Nanoparticle
NSCLC: Non-small cell lung cancer
RCC: Renal cell carcinoma
AEs: Adverse events
CR: Complete responses
PR: Partial responses
LNAs: Locked nucleic acids
UTR: Untranslated region
SOCS1: Suppressor of cytokine signaling-1
cEt: S-constrained ethyl
NDUFA4: NADH dehydrogenase (ubiquinone) 1 alpha subcomplex 4
PTBP1: Polypyrimidine tract binding protein 1
